# Impact of Plasticizer on the Intestinal Epithelial Integrity and Tissue-Repairing Ability within Cells in the Proximity of the Human Gut Microbiome

**DOI:** 10.3390/ijerph20032152

**Published:** 2023-01-25

**Authors:** Tim-Fat Shum, Liwen Wang, Jiachi Chiou

**Affiliations:** 1 Department of Food Science and Nutrition, The Hong Kong Polytechnic University, Hung Hom, Kowloon, Hong Kong, China; 2 Research Institute for Future Food, The Hong Kong Polytechnic University, Hung Hom, Kowloon, Hong Kong, China

**Keywords:** phthalate plasticizer, di(2-ethylhexyl) phthalate (DEHP), di-iso-nonyl phthalate (DINP), gut microbiota, intestinal epithelial cell, macrophage, leaky gut, dysbiosis, intestinal inflammation

## Abstract

Toxicological research into the impact of plasticizer on different organs has been reported in the past few decades, while their effects on shifting the gut microbiota and immune cells homeostasis in zebrafish were only studied recently. However, studies on the impact of plasticizer on human gut microbiota are scarce. In this study, we co-incubated healthy human fecal microbiota with different concentrations of Di(2-ethylhexyl) phthalate (DEHP) and di-iso-nonyl phthalate (DINP), analyzed microbial composition by 16S rDNA sequencing, and compared the influence of their derived microbiomes on the human enterocyte (HT-29) and murine macrophage (RAW264.7) cell lines. Microbial diversity is reduced by DEHP treatment in a dose-dependent manner. DEHP treatment reduced the phyla Firmicutes/Bacteroidetes ratio, while DINP treatment promoted Proteobacteria. Expressions of tight/adherens junction genes in HT-29 and anti-inflammatory genes in RAW264.7 were down-regulated by plasticizer-co-incubated microbiota derived metabolites. Overall, it is observed that selected plasticizers at high dosages can induce compositional changes in human microbiota. Metabolites from such altered microbiota could affect the tight junction integrity of the intestinal epithelium and upset macrophage differentiation homeostasis in proximity. Chronic exposure to these plasticizers may promote risks of dysbiosis, leaky gut or the exacerbation of intestinal inflammation.

## 1. Introduction

Phthalates are one of the major groups of environmental contaminants with global occurrence. They have been ubiquitously found in human urine and blood, posing potential health issues [1]. Di(2-ethylhexyl) phthalate (DEHP) and di-iso-nonyl phthalate (DINP) are widely used as plasticizers to soften poly-vinyl chloride (PVC) plastics for flexibility [2]. The primary concern is that DEHP can be metabolized into different toxic metabolites such as Di-n-octyl phthalate (DnOP) and mono-(2-ethylhexyl) phthalate (MEHP) when it enters the human body. Studies have shown that it can induce reproductive toxicity and endocrine disruption. It is best known as an endocrine disruptor that disrupts the regulation and action of thyroid hormones [3]. In addition, DEHP and its metabolites have the potential to induce testicular, ovarian, renal, neuro, and cardiovascular toxicity [4,5,6]. Both occupational and environmental routes of exposure to DEHP are of concern for human health [7]. Due to the toxic effect of DEHP, another phthalate, DINP, is used as an alternative to DEHP during PVC production. It is suggested that DINP has lower cytotoxicity and carcinogenicity compared with DEHP; however, some studies have demonstrated that DINP can also disrupt immune homeostasis and cause spleen, liver, and kidney damages [1,2]. In addition to the alteration of metabolisms by DEHP/DINP or their derivatives, recent study has demonstrated that DEHP is able to modify the microbiome–gut–immune axis and upset gastrointestinal homeostasis. Transcriptomic and metagenomic analyses reveal that gene expressions of intestine associated T helper cell subsets and tight junction proteins are changed after DEHP exposure in zebrafish [8]; however, studies regarding the effects of plasticizer on human microbiota are relatively limited.

Since oral intake is the major exposure pathway for DEHP and DINP to enter the human body, and their systemic availabilities were verified to be about 7% and 50% in rats, respectively [9,10,11], we hypothesize that such plasticizers can shift the microbial composition of human microbiota, in turn affecting intestinal tight/adherens junction (TJ/AJ) integrity and macrophage (pro-inflammatory M1/tissue repairing M2) differentiation. We adopt a batch culture model, which consists of fermenters simulating the human intestinal ecosystems of colons, to investigate the alteration of microbiota by a wide range of plasticizer concentrations. Fecal microbiota were cultured anaerobically with different concentrations of DEHP and DINP for one day. The resultant microbiota compositions were analyzed by 16S rDNA sequencing. The influence of microbial metabolites from luminal extracts on cultural enterocytes and macrophage was investigated.

## 2. Materials and Methods

### 2.1. Batch Culture and Preparation of Luminal Extracts

**Figure 1:**
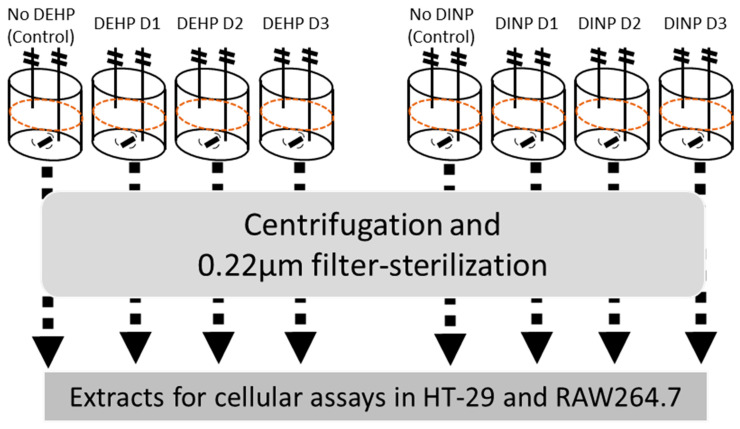
Experimental workflow. After 24 h fermentation, sterilized luminal extracts from fermenters treated with no plasticizer control, DEHP or DINP were used for cell assays in vitro utilizing human intestinal epithelial cell line HT-29 and murine macrophage cell line RAW264.7.

Fecal samples were collected from a healthy donor (male aged 25; non-smoker) who had a normal diet and did not intake any probiotics or antibiotics for at least 2 weeks before sampling day. Fecal material was stored in gas-tight sample bucket with anaerobic sachet (Thermo Fisher, Waltham, MA, USA) and inoculated within 4 h after collection. The final concentration of 10% *w*/*v* of fecal material was homogenized with anaerobic buffer (phosphate buffer: 8.8 g/L K_2_HPO_4_, 6.8 g/L KH_2_PO_4_, 0.1 g/L sodium thioglycolate, and 15 mg/L sodium dithionite) inside a stomacher bag with filter (Seward, West Sussex, UK) for 5 min at 200 rpm. The filtrate portion was inoculated in sterilized Adult Growth Medium (without starch) (ProDigest, Gent, Belgium) of pH 6.9, which mimics the condition of the distal colon [12], with a volume ratio of 1:10. Starting cultural volume in each chamber was at 200 mL. One of the four inoculated chambers was used as no treatment control (D0), while three dosages of DEHP or DINP (Sigma, Saint Louis, MA, USA) were introduced into each treatment cultural chamber (Figure 1). The three concentrations (D1, D2 and D3) of DEHP were set as 1.05, 10.5, and 105 μg/mL, while those of DINP were 0.168, 1.68, and 16.8 μg/mL, respectively, according to possible daily exposures detected from different studies [7,10,13,14,15]. The culture chamber was purged with nitrogen for 10 min after inoculation and maintained at 37 °C for 24 h. Fermenter contents were aliquoted and stored in −20 °C until analysis. Resultant mixtures were subjected to determination of short-chain fatty acids (SCFAs) (acetate, propionate and butyrate) by gas chromatography with flame ionization detector [16,17]. For the preparation of luminal extract, the resultant fermentation culture was defrosted from −20 °C and centrifuged at 15,000× *g* for 30 min at 4 °C. Supernatant from centrifugation was filter-sterilized by 0.22 μm syringe filter. Filtrate was then aliquoted into 2 mL micro-centrifuge tubes and stored at −20 °C as luminal extract.

### 2.2. DNA Extraction, 16s rDNA Sequencing and Microbial Composition Analysis

Samples from all chambers were centrifuged at 12,000 rpm for 5 min. Genomic DNA was extracted from the pellet using stool DNA extraction kit (Tiangen, Beijing, China). Extracted DNA concentration was determined by NanoDrop 2000 (Thermo Fisher, Waltham, MA, USA). Bacterial 16S rDNA sequencing of V3-V4 region (336F-806R) was conducted by MajorBio Ltd. (Shanghai, China). Microbiota analysis was performed using MajorBio analysis platform.

### 2.3. Cell Culture and Viability Assay

Human intestinal epithelial cell line HT-29 (ATCC, HTB-38) and murine macrophage RAW264.7 (ATCC, TIB-71) were maintained in complete medium (Dulbecco’s Modified Eagle’s Medium (DMEM) with 10% fetal bovine serum (FBS) in a 5% CO_2_, 37 °C humidified incubator. HT-29 enterocyte monolayers were differentiated in 24-well cell culture plate starting at 2 × 10^4^ cell/well for 12–14 days before treatment or viability assay. RAW264.7 was seeded at 20 × 10^4^ cell/well in a 24-well culture plate 24 h before treatment or viability assay.

The viabilities of cells after incubating with a range of luminal extracts were determined by 3-(4,5-dimethylthiazol-2-yl)-2,5-diphenyltetrazolium bromide (MTT) assay. Treatment media containing different concentrations of luminal extracts from each chamber were incubated with differentiated HT-29 monolayer or RAW264.7 cells for 24 h. Cells were washed by phosphate-buffered saline (PBS) pH 7.3 and incubated with 0.5 mg/mL MTT for 1–1.5 h in triplicate. Luminal extract concentrations (*v*/*v*%) were employed as treatment percentage for gene expression assay, which preserved >90% cell viability, mimicking a sub-lethal chronic exposure to each cell-line (Supplementary Figure S1).

### 2.4. RNA Extraction and Reverse Transcription Quantitative PCR

HT-29 and RAW264.7 cells were treated with 1 mL of 10% and 5% *v*/*v* luminal extracts, respectively, in complete medium for 24 h inside a cell culture incubator. Total RNA was extracted using RNAiso PLUS (Takara, Shiga, Japan). RNA concentration was determined by NanoDrop spectrophotometer. Complementary DNA (cDNA) was reverse transcribed using PrimeScript reverse transcription kit (Takara). Quantitative PCR was performed on QuantStudio 7 Flex Real-Time PCR System (Thermo Fisher, Waltham, MA, USA) with TB Green Premix Ex Taq II (Takara) reagent. Relative quantification of target gene expression was calculated by 2^−ΔΔCt^. Ribosomal protein lateral stalk subunit P0 (*RPLP0*) and hydroxymethylbilane synthase (*HMBS*) were used as housekeeping genes in HT-29 and RAW264.7, respectively. Primers for specific mRNA amplification, including TJ/AJ reactive oxygen species (ROS) related and pro-/anti-inflammatory mediator genes, are listed in the Supplementary Tables S1 and S2 [18,19].

### 2.5. Western Blot

Western blot was employed for the comparison of protein expressions of tight junctions and adherens junctions after fermenter extract treatment. HT-29 cells were seeded at 2 × 10^4^ cell/well in a 24-well plate. HT-29 was incubated with 20% extracts for 48 h prior to protein quantification. Cells were washed with PBS and then incubated in RIPA buffer (Millipore, Burlington, MA, USA) with protease inhibitor cocktail for 2 h at 4 °C. Total protein was obtained in supernatant after centrifugation at 20,000× *g* for 10 min. Protein samples were stored at −80 °C before quantification by BCA assay.

A total of 20 μg of protein from each sample was mixed with sample buffer and heated at 95 °C for 5 min before loading to SDS-PAGE gel. Western blot transfer was carried out with 80 V for 90 min at 4 °C. PVDF (GE healthcare) after transferring was blocked with blocking solution (5% skimmed milk in Tris buffered saline with 0.1% Tween 20 (TBST)) for 2 h, and then incubated with primary antibody overnight at 4 °C. Primary antibodies used for this study include mouse monoclonal antibody against β-actin (Sigma, Saint Louis, MA, USA), and rabbit polyclonal antibodies against ZO-1, E-cadherin, occluding, F11R, claudin-4 and histone-H3 (ABclonal, Woburn, MA, USA). Secondary antibodies, horseradish peroxidase-conjugated goat anti-rabbit IgG (H + L) (Novus Biologicals, Centennial, CO, USA) and goat anti-mouse IgG (H + L) (Invitrogen, Waltham, MA, USA), were used for chemiluminescent signal detection during image capture.

Densitometry of chemiluminescent signal was analyzed using Image J. Relative expression of target protein was calculated by dividing its signal to β-actin signal. Normalized expression of target proteins in each sample was generated by dividing the relative expression by average relative expression in control fermenter extract group. Target protein expression in the control fermenter treatment group was normalized as 1.

### 2.6. Immunofluorescence

Immunofluorescence was used for the validation of changes in claudin-2 expression in HT-29 cells. HT-29 cells were cultured and co-incubated with luminal extract as described in the RT-qPCR section. Cells were washed thrice with PBS and fixed by 4% PFA for 10 min. Followed by the removal of PFA and three PBS washes, cells were permeabilized with 0.1% Triton X-100 for 10 min. Permeabilized cells were washed with PBS again, and then blocked by blocking buffer (10% FBS and 1% BSA in PBS) for 30 min. Cells were incubated with rabbit polyclonal anti-claudin-2 (ABclonal, Woburn, MA, USA) in blocking buffer overnight at 4 °C. Following three PBST (0.1% Tween 20 in PBS) washes, cells were incubated with goat anti-rabbit IgG (H + L) Alexa Fluor^TM^ 488 (Invitrogen, Waltham, MA, USA) for 2 h at 4 °C. The coverslip was removed from the well and inversely placed onto one drop of fluoroshield mounting medium with DAPI (Abcam, Cambridge, UK) on glass slide, and then sealed with nail polish. Fluorescence signals were captured by ZEISS Axio Vert. A1 with objective magnification of 40×. Width of images was 330 microns. Exposure time for FITC and DAPI channels were 300 ms and 150 ms, respectively. Duplicate staining was performed for each treatment. Fluorescence intensities were analyzed using Image J. Fluorescent intensity was averaged from three random areas of each image. Relative expression of claudin-2 was calculated by dividing claudin-2 signal by DAPI signal. Normalized expression of claudin-2 in each sample was generated by dividing each relative expression by average relative expression of control fermenter extract group. Claudin-2 expression in control fermenter treatment group was normalized as 1.

### 2.7. Statistical Analysis

Statistical comparisons were performed using GraphPad Prism 5. All results are shown as mean +/- SEM from the replicates. Statistical analysis, *t*-test, was used with 95% confidence.

## 3. Results

### 3.1. DEHP and DINP Altered the Compositions of Gut Microbiota

**Figure 2:**
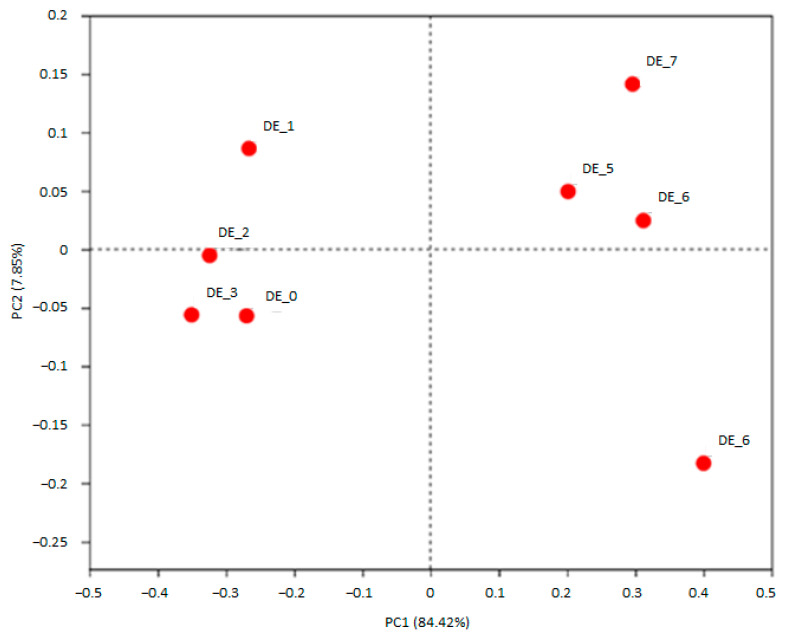
PCoA plot showing no DEHP control, DEHP-D1, DEHP-D2 and DEHP-D3 microbiota compositions at 0 h (DE_0 to DE_3) and 24 h (DE_4 to DE_7).

**Figure 3:**
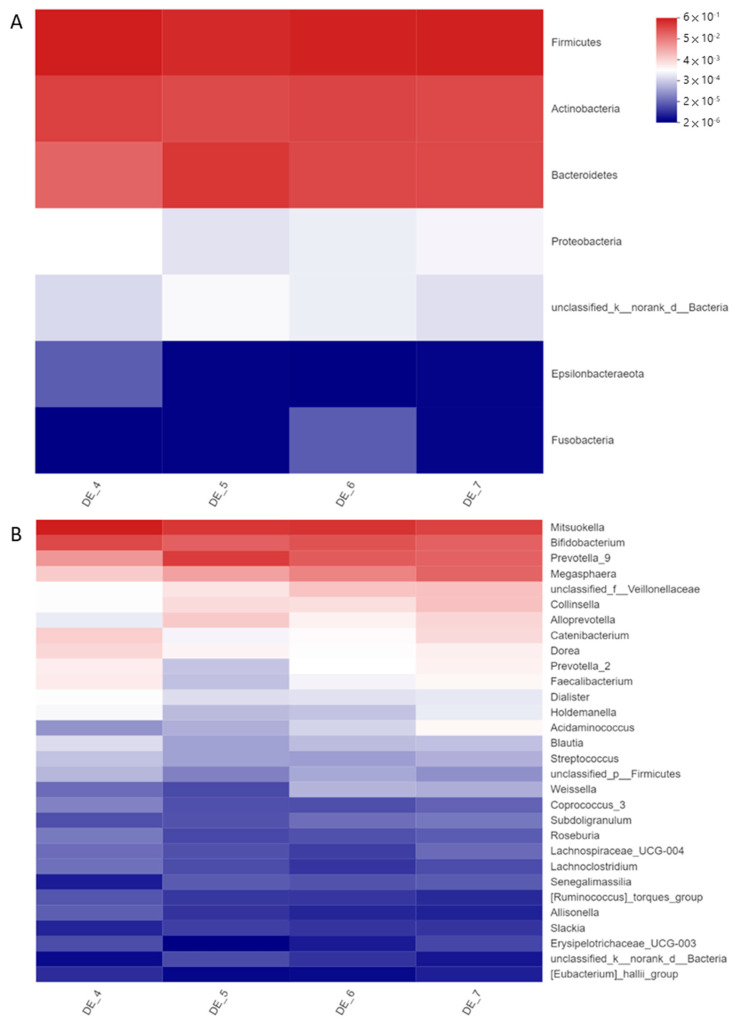
Relative abundance at (**A**) phylum level and (**B**) genus level in resultant microbiota of no DEHP control (DE_4), DEHP-D1 (DE_5), DEHP-D2 (DE_6) and DEHP-D3 (DE_7) fermenters.

A total of 315,669 qualified reads of 16S rDNA in eight resultant fermenters were used for detailed diversity and taxonomy analysis. Shannon indices of DEHP-treated microbiota were increased, while Simpson’s indices were reduced, whereas phylogenetic distances (pd) were all reduced in DEHP-D1 to DEHP-D3 microbiota comparing with no DEHP control (Table 1). Microbial diversity and richness were slightly increased in DEHP-treated microbiota; however, the phylogenetic distance among these features became smaller. The microbiota compositions at the beginning of the DEHP experiment were clustered together. After 24 h of incubation, DEHP containing fermenters were clustered away from the control chamber (Figure 2). Microbial compositions of all DEHP-treated fermenters deviated from the no treatment control. Similar to the observation in previous studies of human gut microbiota, the most abundant phyla in all the fermenters were Firmicutes, Actinobacteria and Bacteroidetes [20]. Proteobacteria and Epsilonbacteraeota were found to be more abundant in DEHP-treated fermenters (Figure 3A). Compared to the control fermenter without treatment, DEHP-treated fermenters had enriched *Alloprevotella*, *Collinsella*, *Megasphaera*, *Prevotella_9* and *unclassified_f__Veillonellaceae* at genus level (Figure 3B). On the other hand, the relative abundance of genera *Bifidobacterium*, *Catenibacterium*, *Dorea*, *Faecalibacterium*, *Holdemanella*, *Mitsuokella* and *Prevotella_2* were all reduced when DEHP was present in the fermentation mixture (Figure 3B).

**Figure 4:**
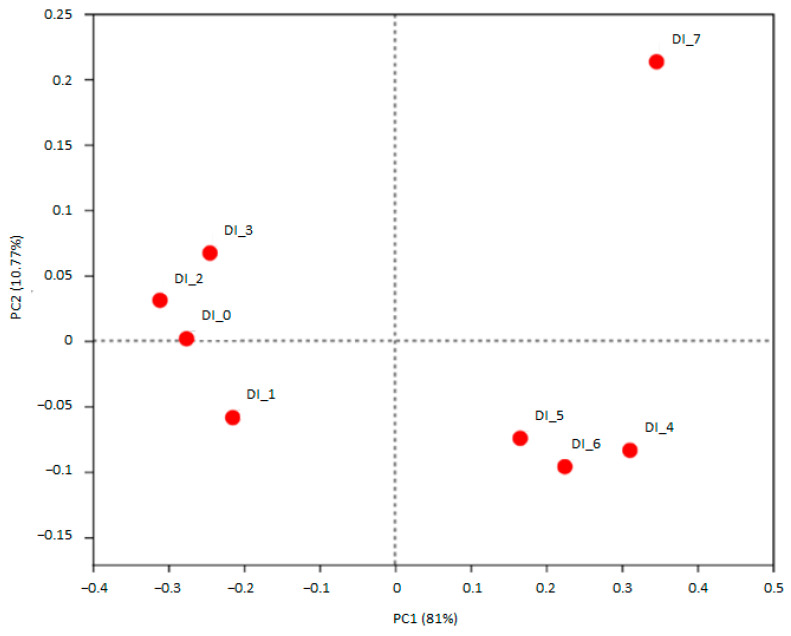
PCoA plot showing no DINP control, DINP-D1, DINP-D2 and DINP-D3 microbiota compositions at 0 h (DI_0 to DI_3) and 24 h (DI_4 to DI_7).

**Figure 5:**
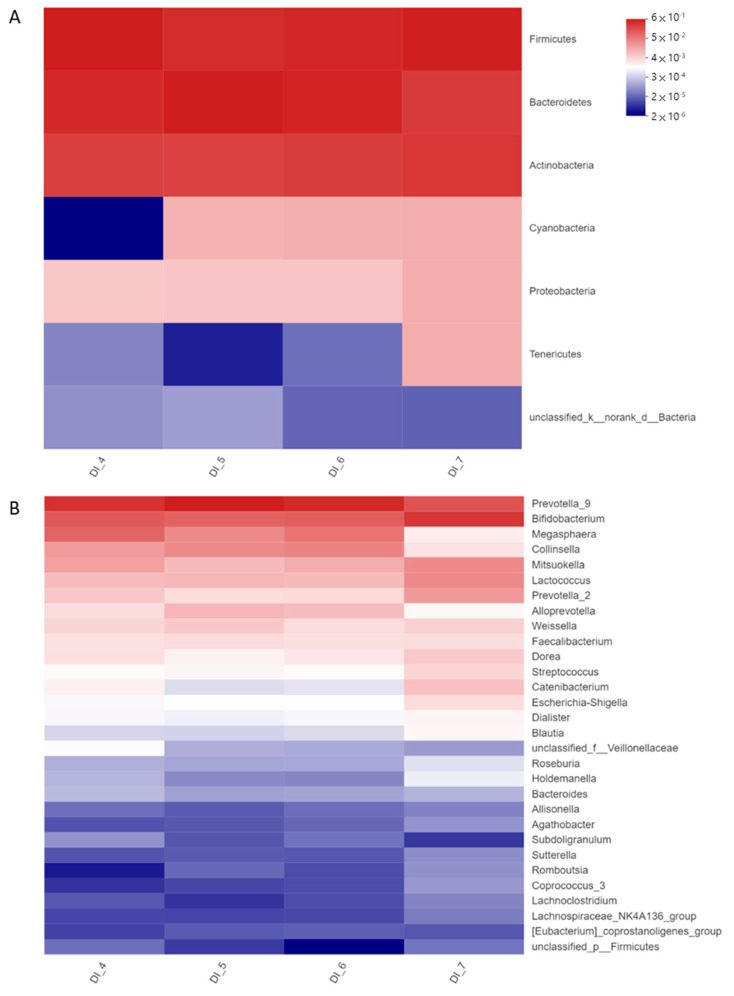
Relative abundance at (**A**) phylum level and (**B**) genus level in resultant microbiota of no DINP control (DI_4), DINP-D1 (DI_5), DINP-D2 (DI_6) and DINP-D3 (DI_7) fermenters.

The microbiota co-incubated with DINP-D1 and DINP-D2 showed fluctuated yet similar indices of Shannon, Simpson and pd compared with the control. At the highest dosage group (16.8 μg/mL), the Shannon index was increased slightly while Simpson and pd remained stable (Table 2). DINP-D3 treated microbiota had higher feature diversity and richness, though they have similar phylogenetic distance. In the fermenters with DINP, starting microbiota compositions were clustered together. The taxonomy of no DINP control, DINP-D1 and DINP-D2 fermenters after 24 h incubation were clustered, while the DINP-D3 community shifted away from all the others in the PCoA plot (Figure 4). Microbial composition in the DINP-D3 fermenter was deviated from no DINP control, DINP-D1 and DINP-D2. Dose-dependent increases in the relative abundance of phyla Actinobacteria, Cyanobacteria and Proteobacteria were detected (Figure 5A). At the Genus level, the relative abundance of *Streptococcus* and *Lactococcus* were increased in DINP-D1 and DINP-D3 fermenters, while they stayed relatively stable in DINP-D2. Moreover, the relative abundance of *Blautia*, *Catenibacterium*, *Dorea*, *Escherichia-Shigella*, *Holdemanella* and *Prevotella_2* in the DINP-D3 fermenter were around two-fold that in the control fermenter. Though increases in *Prevotella_2* were observed in the DINP-D3 community, the relative abundance of *Prevotella_9* was reduced by about 40% compared with control. Finally, a dramatic shrink in *Megasphaera* abundance was detected in the DINP-D3 community (Figure 3B).

### 3.2. Luminal Extracts from Plasticizer-Treated Microbiota Altered Gene Expressions in HT-29 and RAW264.7 Cells

**Figure 6:**
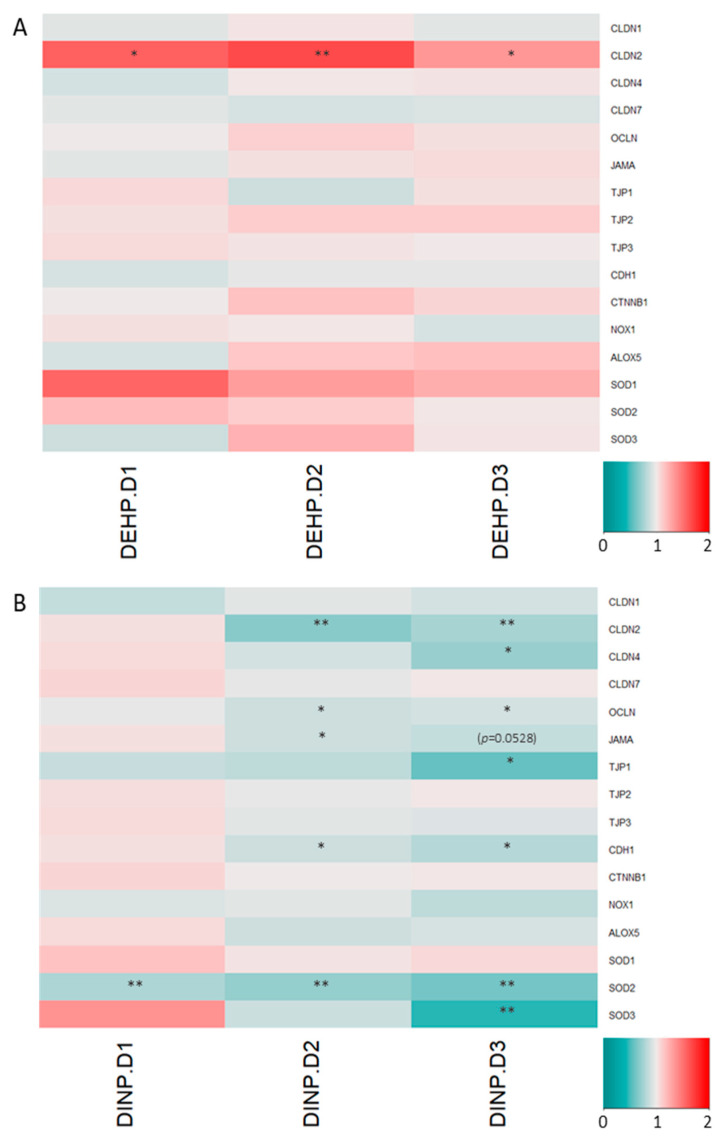
Relative quantification of TJ/AJ- and ROS-related gene mRNA levels in HT-29 after treatment of (**A**) DEHP experiment fermenters’ extracts and (**B**) DINP experiment fermenters’ extracts. Expression in cells treated with luminal extracts from no plasticizer fermenter was normalized as 1. *t*-test, * p < 0.05, and ** p < 0.01.

Expressions of major tight junction and adherens junction proteins remained similar when HT-29 was treated with luminal extracts from the DEHP fermenter. However, HT-29 *CLDN2* expressions in all DEHP-treated fermenter extract treatment groups were enhanced by >35% (Figure 6A). Several trans-membrane TJ/AJ and their intracellular adaptor proteins’ genes, including *CLDN2*, *CLDN4*, *OCLN*, *TJP1*, *CDH1*, *SOD2* and *SOD3*, were down-regulated in HT-29 treated with high DINP-dosed fermenter luminal extracts. Significant reductions in adherens junction *CDH1* mRNA levels were observed in DINP-D2 and DINP-D3 fermenter luminal extracts treated HT-29 by 12% and 20%, respectively, (Figure 6B). Tight junction *OCLN* expression levels were dropped by 10% when HT-29 was exposed to DINP-D2 or DINP-D3 extracts. Decreases in expressions of *CLDN2* and *CLDN4* were found in DINP-D3 luminal extract treated HT-29 (by 24% and 19%, respectively). Around a 15% drop in *JAMA* expression was also observed in the HT-29 treated with the highest DINP (*p* = 0.0528). *TJP1* coding for ZO-1 was down-regulated by more than 40% in the HT-29 treated with the same dose. For expressions of ROS related gene at the highest DINP-dosed extract treatment, superoxide dismutase-2 and -3 (*SOD2* and *SOD3*) gene expressions were diminished by 7% and 21%, respectively.

**Figure 7:**
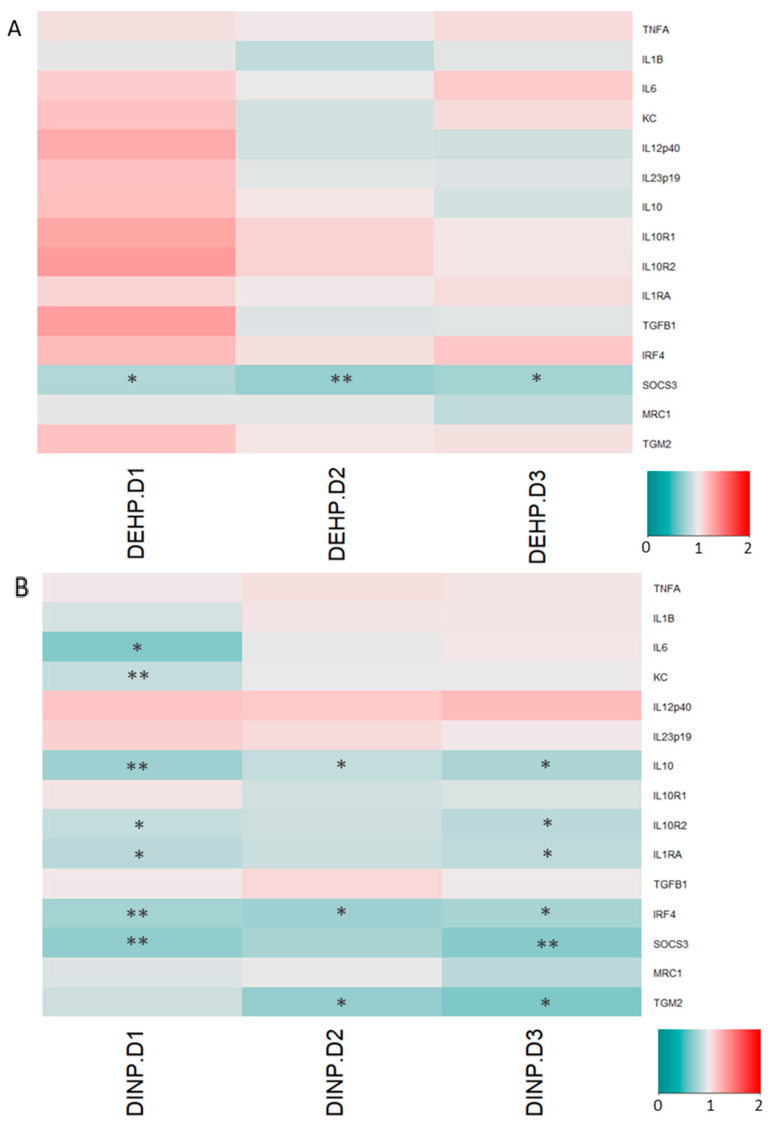
Relative quantification of cytokine mRNA levels in RAW264.7 after treatment of extracts from (**A**) DEHP fermenters and (**B**) DINP fermenters. Expression levels of mRNA in cells treated with luminal extracts from no plasticizer fermenter were normalized as 1. *t*-test, the data were produced in triplicate and *t*-test was used for statistical analysis. * p < 0.05, and ** p < 0.01.

Cytokines and cytokine receptors in RAW264.7 cells treated with different DEHP dosed-luminal extracts remained similar. Among the selected alternative activated M2 genes, the expression of the suppressor of cytokine signaling 3 (*SOCS3*) was reduced in macrophage treated with DEHP fermenter extracts (Figure 7A).

Anti-inflammatory mediators in RAW264.7 were downregulated, while pro-inflammatory cytokine expressions remained stable after exposure to DINP-altered microbiota extracts. IL-1 receptor antagonist (*IL1RA*) mRNA levels in RAW264.7 cells were decreased by about 17% in DINP-D1 and DINP-D3 treatment groups. More than 15% of drops in *IL10* (encoding interleukin-10) and *IL10R2* (encoding IL-10 receptor 2) expressions were detected in all DINP-dosed luminal extract treated RAW264.7. The microbiome of the DINP-treated fermenter induced suppressions of *IRF4* (encoding interferon regulatory factor-4) and *SOCS3*. Significant downregulations of Transglutaminase-2 (*TGM2*) transcript were detected in DINP-D2 and DINP-D3 extracts treated RAW264.7 (Figure 7B).

### 3.3. Microbiota Metabolites from Fermenter Exposed to High-Dose DINP Reduced Protein Expressions of E-Cadherin, JAM-A and Claudin-4 in HT-29

**Figure 8:**
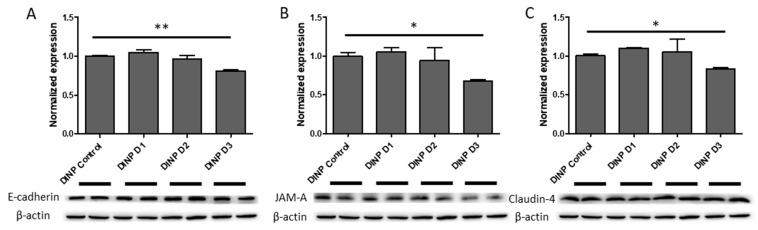
Expressions of (**A**) E-cadherin, (**B**) JAM-A and (**C**) Claudin-4 in HT-29 after luminal extract treatments. Expression levels of protein in cells treated with luminal extracts from no plasticizer fermenter were normalized as 1. The data were produced in triplicate and *t*-test was used for statistical analysis. * *p* < 0.05, and ** *p* < 0.01.

The messenger RNA coding for E-cadherin in HT-29 was reduced by 20% after the treatment of DINP-D3 extract compared to DINP-D0 control extracts. Consistently, E-cadherin was also reduced by almost 20% in HT-29 at protein level (Figure 8A). Claudin-4 was also reduced by 17% after DINP-D3 incubation (Figure 8C). Consistent reductions of both *JAMA* mRNA and its protein were observed after the treatment of DINP-co-incubated fermenter extracts. From Western blot, more than 30% of JAM-A proteins were lost after DINP-D3 extract treatment (Figure 8B). Although reductions of *TJP1* and *OCLN* mRNA levels in HT-29 treated with DINP-dosed fermenter extracts were observed, proteins levels of ZO-1 and occludin in HT-29 remained similar after the treatment (Supplementary Figure S2A,B).

### 3.4. DEHP Fermenter Luminal Extracts Promoted Claudin-2 Expression in HT-29, whereas Reduction of this Protein Was Observed after Treatment of DINP Fermenter Extracts

**Figure 9:**
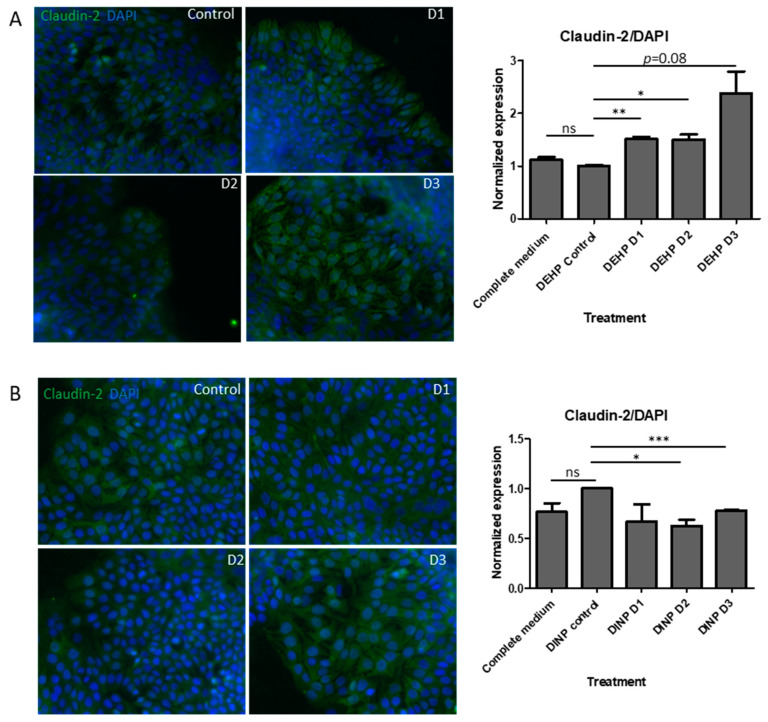
Claudin-2 expression in HT-29 after (**A**) DEHP and (**B**) DINP experimental luminal extract treatments. Expression of Claudin-2 in cells treated with luminal extracts from no plasticizer fermenter was normalized as 1. Image width = 330 µm. *t*-test, ns: not significant, * p < 0.05, ** p < 0.01 and *** p < 0.005.

Expressions of claudin-2 also translated from mRNA level to protein level. After DEHP-D2 and DEHP-D3 extracts treatments, cladudin-2 in HT-29 was increased by 50%. The average enrichment of cladin-2 was up to 2.4-fold that of the fermenter extract without DEHP treatment (*p* = 0.08) (Figure 9A). Consistent with the percentage drops obtained in *CLDN2* qPCR results, claudin-2 in HT-29 treated with DINP-D2 and DINP-D3 extracts was down-regulated by 30% and 20% (Figure 9B).

## 4. Discussion

The beta-diversity PCoA analysis showed that all starting communities in DEHP or DINP experiments were clustered together, indicating they have a relatively similar microbiota baseline to start with. After 24 h of incubation, the microbial communities of the DEHP-treated fermenter (DE_5, DE_6 and DE_7) were all clustered away from that of the control fermenter (DE_4). By contrast, only the microbiota composition of the DINP-D3 fermenter (DI_7) was deviated from that of the fermenters of control, DINP-D1 and DINP-D2. These results suggested all three DEHP treatments and the highest DINP dosage significantly altered the composition of baseline microbiota. Studies have revealed that inflammatory bowel disease (IBDs) patients have a microbiota with on average 25% less bacterial abundance and diversity [21,22]. Though higher Shannon indices are recorded in DEHP-co-incubated communities, those communities exhibit a dosage-dependent decrease in community functional phylogenetic distance. In a DINP experiment, alpha diversity indices fluctuate and similar biodiversity is observed between no DINP control and DINP-contaminated communities.

Microplastics (MPs) are one of the carriers of plasticizers and biofilm can be readily formed on them due to their large surface area. Chung et al. indicated that major biofilm colonizers are from phyla Proteobacteria, followed by Bacteroidetes, Firmicutes, Acidobacteria and Actinobacteria [23]. Not only MPs but also plasticizers could also contribute to changing microbiota compositions. Studies on the microbiota composition of zebrafish treated with DEHP have revealed that such plasticizers can modify the community by reducing the overall diversity and enriching phyla Fusobacteria, Bacteroidetes and Verrucomicrobia [8]. Consistent observation was obtained in our DEHP experiment on human microbiota, which had increases of phylum Bacteroidetes in all DEHP-containing fermenters. On the other hand, a dosage-dependent growth of phylum Proteobacteria is detected in DINP treatment groups. The enrichment of this phylum has long been described in inflammatory diseases and dysbiosis, and similar observations have been reported in comparative studies related to gastrointestinal inflammation [24].

Several genera are enriched in the DHEP-treated fermenter, including *Alloprevotella*, *Collinsella*, *Megasphaera* and *Prevotella*. Studies have found that *Prevotella* were related to pro-inflammatory responses exacerbating colitis in DSS-induced animal models by impairing the repair mechanisms of the mucosa [25,26]. Clinically, *Prevotella* is reduced in the microbiota of Crohn’s disease (CD) patients in remission, receiving exclusive enteral nutrition [27]. The relative abundance of *Alloprevotella* and *Prevotella_9* was enhanced in all DEHP-treated microbiota, whereas a decrease in *Prevotella_2* was observed simultaneously. Collectively, the increase of *Prevotellaceae* is contributed by the major expansion of *Prevotella_9*. *Collinsella* was found enriched in colitis patients’ microbiota [28,29]. Interestingly, DEHP also favored the expansion of *Collinsella*, which was the only known taxon under the family *Coriobacteriaceae* to date. This family is more commonly found in CD patients’ mucosa and submucosa microbiomes [30]. In the DINP experiment, *Escherichia-Shigella* and *Streptococcus* accounted for the most abundance of the family *Enterobacteriaceae* and *Streptococcaceae*. These genera have been detected more abundantly in patients with inflammatory diseases [24,28,31]. In addition, a study has shown that CD post-operative recurrence is associated with the relative abundance of *Streptococcus* [29].

Decreases in the relative abundance of *Bifidobacterium* were observed in all DEHP-treated microbiota, with the greatest drop observed in DEHP-D3 fermenter. A reduction in *Bifidobacterium* has been detected in the onset of CD patients’ microbiota, and its supplementation had been demonstrated to improve intestinal barrier function, leading to a lower plasma endotoxin level [24,28,32]. SCFAs, including acetate, propionate, and butyrate, promote intestinal barrier function and induce antigen presenting cells (APCs) such as dendritic cells and macrophages. SCFAs, particularly butyrate, can stimulate regulatory T cell differentiation to maintain immune homeostasis [33,34,35]. Though the concentration of SCFAs remained similar in each experiment set (data not shown), the alterations of several SCFAs producers’ abundance were observed in the microbiota treated with plasticizer. Genera *Blautia*, *Dorea*, *Lachnospira*, *Roseburia* and *Faecalibacterium* are less abundant in the microbiota of CD patients [24,29,33]. *Faecalibacterium prausnitzii* is one of *Faecalibacterium* spp. that produces SCFAs and regulates immune cells by acting on mentioned APCs. Its loss of abundance in the gut microbiota has been frequently described as a marker of IBDs. Interestingly, DEHP-treated microbiota has lower *Dorea* and *Faecalibacterium* abundance, implying the exposure of DEHP may be one of the various factors promoting the occurrence of IBD.

Intestinal epithelial cells beneath mucus layers contribute to the formation of a barrier separating bacteria in the gut lumen from blood vessels. Epithelium integrity is crucial in preventing external antigens from contacting immune cells, trigging inflammation responses. Influences from the dynamic changes of microbiota affect claudin remodeling, which occurs within several days due to the short life span of IECs [36]. TJ and AJ proteins are devoted to constructing the epithelial barrier, which connect epithelial cells together and limits solute passages through the paracellular space. Claudin-1, claudin-4 and claudin-7, which are normally expressed in the colon for TJ sealing, are observed reduced in acute colitis, CD and colitis patients [37,38,39]. The deterioration of TJ proteins such as occludin and JAM-A have also been shown to upset barrier functions, leading to leaky-gut and endotoxemia [39,40]. AJ E-cadherin and its adapter protein β-catenin support junction stability. Moreover, ZO-1, ZO-2 and ZO-3 (encoded by *TJP1*, *TJP2* and *TJP3*) are major intracellular adapter proteins interacting both transmembrane TJ proteins and cytoskeletal proteins [39]. Though DEHP alters microbiota towards dysbiosis, mRNA levels of most selected target genes encoding TJ/AJ sealing proteins in this study remain stable in HT-29 cells. Nonetheless, mRNA levels of *CLDN4*, *OCLN*, *JAMA*, *TJP1* and *CDH1* are down-regulated (by 10–43%) in DINP-D3 luminal extract treated HT-29 cells. A significant reduction (12%) in expressions of *OCLN*, *JAMA* and *CDH1* is observed even when the cells are incubated with DINP-D2 luminal extracts. Western blot assays verify that E-cadherin, JAM-A and claudin-4 proteins are significantly reduced after DINP-D3 fermenter treatment.

Apart from proteins promoting paracellular integrity, participating in nutrient absorption, cation-leaky pore-forming claudin-2 expression is localized in the duodenum and ileum. However, studies have demonstrated that claudin-2 can also increase intestinal epithelial cell paracellular permeability, leading to diarrhea, and is highly expressed in colitis patients’ colons [41]. Enhanced claudin-2 expression can be induced by microbial metabolites, and may be intended to result in an osmotic imbalance between the epithelium to remove unwanted contents such as pathogens and their products from the lumen [42,43]. Claudin-2 is enhanced by 35–67% in HT-29 treated with all DEHP-treated fermenters extracts. Interestingly, down-regulations in both mRNA and protein of claudin-2 in DINP-D2/DINP-D3 extracts treated HT-29 are detected. This may be due to the enrichment of taxon *Bifidobacterium* in DINP-containing fermenters [44].

Apart from TJ and AJ gene expressions, antioxidant and antioxidant enzymes have been demonstrated in reducing the severities of dextran sulfate sodium (DSS)-/trinitrobenzene sulfonic acid (TNBS)-induced ulcerative colitis in a murine animal model [45,46]. High oxidative stress in inflamed tissues can be observed and such status is considered as one of the pathogeneses of colitis [47]. Several types of SODs produced by cells catalyze the disproportionation of reactive oxygen species superoxide ions to oxygen and less harmful hydrogen peroxide. Dismutations of superoxide ions by SODs are drastically reduced in the experimental colitis animal, and SOD activity-elevating antioxidant treatment is found to be effective in relieving such disease [45]. In this study, slightly increased mRNA levels of *SOD1* were observed, whereas gene expressions of *SOD2* and *SOD3* in high dosage DINP-treated HT-29 were reduced by about 40% and 55%, respectively. The three SOD enzymes are located in different regions within cells and their expressions are regulated by various transcriptional factors, in which SOD1 has the most constitutive expression. Therefore, it is not surprising that the *SOD1* mRNA shows it is least affected by the DINP treatment [48]. The relationship between the altered microbial taxa and expressions of SODs remains to be elucidated in future study.

Most pro-inflammatory cytokines (*TNFA*, *IL1B*, *IL6*, *KC*, *IL12p40* and *IL23p19*) expressions remained stable after the treatment of DEHP or DINP co-incubated microbial extracts. By contrast, M2 alternative activated macrophages participate in the repairing of inflammatory damages brought by the constant stimulation of luminal antigens. The expression of *IL1RA*, encoding for IL-1 receptor competitive ligand which is one of the M2-polarization markers, was reduced in macrophages treated with different dosages of DINP fermenter extracts. On the other hand, efferocytosis enzyme transglutaminase (TGM2), another M2 expressed gene which is shared by both human and murine macrophages, was also diminished in those cells [49].

The IL-12/IL-10 ratio is a general indicator of M1/M2 polarization. The IL-10 signaling axis is an important regulator in colitis. The deletion of IL-10 could lead to spontaneous colitis. Moreover, the deletion of macrophage-specific IL-10R1 worsens the disease severity in the DSS-induced colitis animal model, the observation of which is consistent with the IL-10 or IL-10R1 knot-out mice [50]. *IL10* and *IL10R2* expressions are downregulated in macrophages treated with extracts from all DINP-treated fermenters. Except for autocrine stimulation, a significant downregulation of *SOCS3* is observed in almost all plasticizer contaminated microbial extract-treated macrophages in this study. Enhanced SOCS3 signaled by IL-10 promotes the alternative activation of macrophages, inhibiting nitric oxide synthesis and blocking inflammatory cytokine expression [51]. The IFN-γ- and LPS-stimulated signal transducer and activator of the transcription-1 (STAT1) pathway has been demonstrated to be prolonged in SOCS3 deficient macrophages [52]. Pro-inflammatory signaling may be maintained with the lack of SOCS3.

Macrophage alters intracellular signaling according to different extracellular stimulants and responds by expressing different genes with specific functions. Transcription factors in M2-polarized macrophage have been intensively studied to identify treatment targets for autoimmune diseases in recent years. The mRNA levels of *IRF4* are reduced in RAW264.7 cells after DINP-fermenter extract treatments. The IRF-4 deficient macrophage is more sensitive to Toll-like-receptor (TLR) ligand stimulation and expresses more proinflammatory cytokines [53]. Induced IRF4 expression promoted M2-polarization and ameliorated LPS-induced M1 cytokine secretion, alleviating DSS-induced colitis in mice [54]. Macrophages may display pro-inflammatory phenotypes with these expression changes.

## 5. Conclusions

Plasticizers can easily be detected in processed or packaged foods. Utilizing the aforementioned human intestinal epithelial and murine macrophage cell lines, here we have showed that the microbial compositions could be modified within one day of high dosage co-incubation with plasticizers towards dysbiosis. This altered microbiota shifts the luminal substances, which can lead to transcriptional changes in cells located at proximity. DEHP reduces the overall microbiota compositional diversity. Metabolites from these communities promote the expression of claudin-2, which may impair epithelial barrier functions. Reductions of major TJ and AJ gene expression (*CLDN2*, *CLDN4*, *OCLN*, *JAMA*, CDH1 and *TJP1*) are observed in HT-29 cells treated with DINP-D2 and/or -D3 extracts. Compared to those treated with health microbial extracts, RAW264.7 cells express less *SOCS3* when they are treated with DEHP-/DINP-contaminated fermentation extracts. DINP-co-incubated microbial extracts further suppresses anti-inflammatory genes *IL10*, *IL10R2* and *IRF4*, which may skew the macrophage differentiation balance. It has been reported that a high concentration of plasticizers is required to exert direct effects on expressions of some tight junction proteins and cytokines in quail and mouse [55,56,57]. By contrast, the exact direct or indirect effect(s) of plasticizers on human enterocyte or macrophages have not been studied. Microbiota modification might be a factor contributing to the imbalance of barrier integrity and immune homeostasis.

Although previous studies have indicated that limited DINP will persist in the organs of rodents, its metabolites absorbed into the human circulatory system, liver and kidney are not all excreted within 48 h [10,11]. In general, the average daily phthalate intake in Asian areas such as Taiwan, Japan and Hong Kong are not as high as the dosages used in the current study [13,14,15]; however, the chronic accumulation of plasticizers within human body through repetitive intakes could be high, and the effect of such exposure remains a concern for health. It is suggested to avoid using too much plastic during food processing in order to limit the exposure to these artificial chemicals.

## Figures and Tables

**Table 1:** Alpha diversity indices of resultant fermenter microbiota in DEHP experiment.

Alpha Diversities	Control	DEHP D1	DEHP D2	DEHP D3
Shannon	1.88	1.91	2.04	2.25
Simpson	0.28	0.22	0.2	0.16
pd	13.24	12.69	12.39	12.07

**Table 2:** Alpha diversity indices of resultant fermenter microbiota in DINP experiment.

Alpha Diversities	No DINP Control	DINP D1	DINP D2	DINP D3
Shannon	2.59	2.44	2.52	2.72
Simpson	0.13	0.17	0.15	0.12
pd	15.53	16.49	16.54	15.53

## Data Availability

Not applicable.

## Supplementary Materials

The following supporting information can be downloaded at: https://www.mdpi.com/article/10.3390/ijerph20032152/s1, Figure S1: Viability of human intestinal epithelial cell line HT-29 and murine macrophage cell line RAW264.7 after treatment of control fermenter extracts; Figure S2: Western blot verification of tight/adherens junction proteins in HT-29 cell after treatments of DINP fermentation extracts; Table S1: Primers sequences for real-time PCR on gene expressions in HT-29; Table S2: Primers sequences for real-time PCR on gene expressions in Raw 264.7; Table S3: Relative quantification (±SEM) of gene expressions in HT-29 after treatment of DEHP fermenter extracts; Table S4: Relative quantification (±SEM) of gene expressions in HT-29 after treatment of DINP fermenter extracts; Table S5: Relative quantification (±SEM) of gene expressions in RAW 264.7 after treatment of DINP fermenter extracts.
