# Supplementary material for: Impact of Plasticizer on the Intestinal Epithelial Integrity and Tissue-Repairing Ability within Cells in the Proximity of the Human Gut Microbiome
Source: Int J Environ Res Public Health. 2023 Jan 25;20(3):2152. doi: 10.3390/ijerph20032152 (PMC9915929; doi:10.3390/ijerph20032152)
Supplement: Supplementary file 1 [file ijerph-20-02152-s001.zip › ijerph-2036494-supplementary.pdf]

Supplementary files

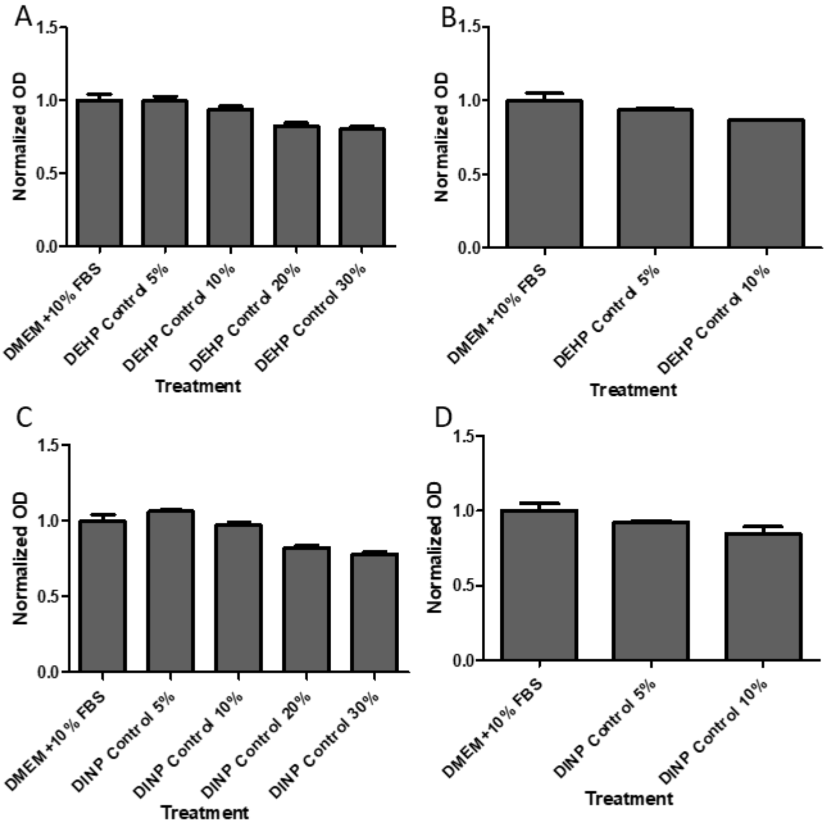

Figure S1. Viability of human intestinal epithelial cell line HT-29 and murine macrophage cell line RAW264.7 after treatment of control fermenter extracts from (**A**, **B**) DEHP experiment and (**C**, **D**) DINP experiment, respectively. Data represents mean with SEM from at least repeats of 3.

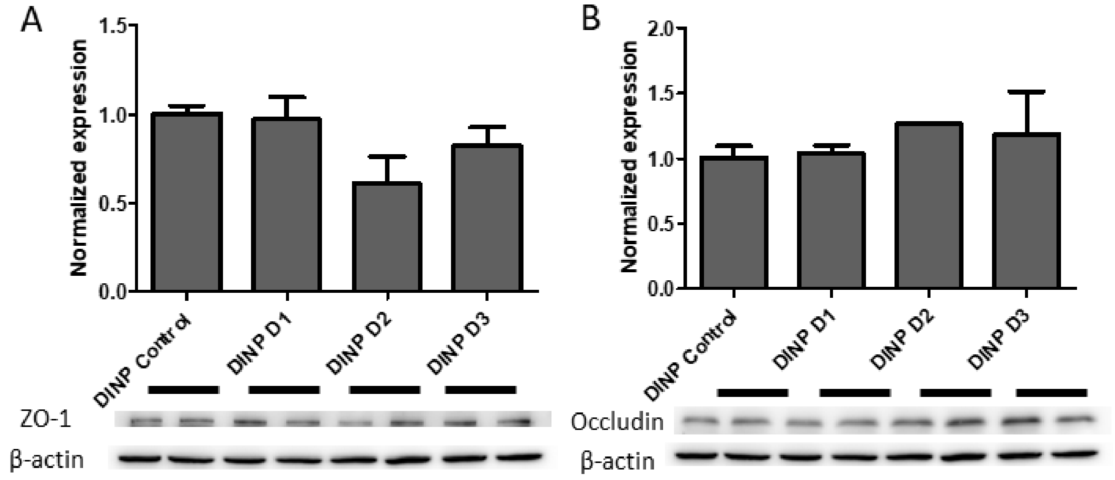

Figure S2. Western blot verification of tight/adherens junction proteins in HT-29 cell after treatments of DINP fermentation extracts. Although not statistically significant, Protein levels of ZO-1 protein levels in DINP-D2 and DINP-D3 extracts' treatment groups were reduced (**A**). Occludin expression was stably maintained in HT-29 after treatment (**B**).

Table S1. Primers for real-time PCR on gene expressions in HT-29.

Gene (*Homo sapiens*)	Protein	Forward sequence (5′-3′)	Reverse sequence (5′-3′)
RPLP0	Ribosomal protein P0	ACTTCCTTAAGATCATCCAACT	TATGAGGCAGCAGTTTCTCCA
OCLN[47]	Occludin	CCAATGTCGAGGAGTGGG	CGCTGCTGTAACGAGGCT
CLDN1	Claudin-1	TGGTGGTTGGCATCCTCCTG	AATTCGTACCTGGCATTGACTGG
CLDN2[48]	Claudin-2	CTCCCTGGCCTGCATTATCTC	ACCTGCTACCGCCACTCTGT
CLDN4	Claudin-4	ATCATCGTGGCTGCTCTGG	ACACCGGCACTATCACCATAA
CLDN7	Claudin-7	TTTCATCGTGGCAGGTCTTG	CTCATACTTAATGTTGGTAGGG
JAMA	JAM-A	GATCACAGCTTCCTATGAGGA	ATGGAGGCACAAGCACGAT
CDH1	E-cadherin	AAGAGGACCAGGACTTTGACTT	CAGCCGCTTTCAGATTTTCATC
CTNNB1	Beta-catenin	GGAATGAGACTGCTGATCTTGG	ATCATCCTGGCGATATCCAAGG
TJP1[1]	ZO-1	CAAGATAGTTTGGCAGCAAGAGATG	ATCAGGGACATTCAATAGCGTAGC
TJP2	ZO-2	GCACAGAATGCAAGGATCGA	GTCTGGAACTCGTGTGCTGG
TJP3	ZO-3	GCGAGAAGCCAGTTTCAAGC	GTCCTGGACACAGTCTCTGCGA
NOX1	NADPH Oxidase 1	TTCCCAGGATTGAAGTGGATGG	GAGGTTGTGGTCTGCACACTGG
ALOX5	Arachidonate 5-lipoxygenase	AGGAGGTCCAGCAAGGGAAC	GATTTGGTTGAGCTGGATGGCA
SOD1	Superoxide dismutase 1	ACAGCAGGCTGTACCAGTGCAG	TTCATGGACCACCAGTGTGC
SOD2	Superoxide dismutase 2	CCAAAGGGGAGTTGCTGGAA	AGGCCTGTTGTTCCTTGCAGTG
SOD3	Superoxide dismutase 3	GCTGGAAAGGTGCCCGACTC	ACCTTGGCGTACATGTCTCGGA

(Adopted from Putt, 2017 [15] and Suzuki, 2011 [16])

Table S2. Primers for real-time PCR on gene expressions in RAW264.7.

Gene (*Mus musculus*)	Protein	Forward sequence (5′-3′)	Reverse sequence (5′-3′)
HMBS	Hydroxymethylbilane synthase	ATGGGCAACTGTACCTGACT	ACCATCTTCTTGCTGAACAG
TNFA	Tumor necrosis factor-α	AAGGGATGAGAAGTTCCCAA	CTTGGTGGTTTGCTACGACGT
IL1B	Interleukin-1 beta	AGGATGAGGACATGAGCACC	GGAGAATATCACTTGTTGGTTG
IL6	Interleukin-6	CTGATGCTGGTGACAACCAC	GCCATTGCACAACTCTTTTCTC
KC	CXCL1	CCGAAGTCATAGCCACACTCAA	CCGTTACTTGGGGACACCTTTTAG
IL12p40	Interleukin-12 p40	AGAGCAGTAGCAGTTCCCCTGA	GGTTTGATGATGTCCCTGATGA
IL23p19	Interleukin-23 p19	CCAGCGGGACATATGAATCTAC	GCAAGCAGAACTGGCTGTTGTC
IL10	Interleukin-10	GCAGGACTTTAAGGGTTACTTGG	AATCGATGACAGCGCCTCAG
IL10R1	Interleukin-10 receptor 1	GTGGATGAAGTGATTCTGACAG	GGTTGCATTCTTTAGTTCTGAG
IL10R2	Interleukin-10 receptor 2	CGTGGAAGACACCATCATTG	CGGAGACACAACTTGAAACTTC
TGFB1	Transforming growth factor beta	CGCAACAACGCCATCTATGA	TATTTCTGGTAGAGTTCCACATG
IL1RA	Interleukin-1 receptor antagonist	GCCTTCAGAATCTGGGATAC	CCAGACTTGGCACAAGACAG
IRF4	Interferon regulatory factor 4	CTCTCAGACTGCCGGCTGCA	CTGGTCCAGGTTGCTAACATCA
SOCS3	Suppressor of cytokine signaling 3	GCTTCGGGACTAGCTCCCCGG	TTGGAGCTGAAGGTCTTGAGGC
MRC1	Mannose Receptor C-Type 1	GAGCAAGCATTTGTTACCTATCAC	CTTCTTCTTCCACCAGGATAGC
TGM2	Transglutaminase 2	GAGCGCCATGGTCAACTGCA	AGCGCCGCAGAATGTCCACA

Table S3. Relative quantification (±SEM) of gene expressions in HT-29 after treatment of DEHP fermenter extracts.

	Relative quantification		
**Gene**	DEHP-D1	DEHP-D2	DEHP-D3
CLDN1	0.9414±0.03942	1.022±0.02363	0.9458±0.04088
CLDN2	1.582±0.1408	1.673±0.05546	1.351±0.08355
CLDN4	0.9009±0.08283	1.012±0.06988	1.035±0.08155
CLDN7	0.9525±0.01062	0.913±0.02419	0.9159±0.03789
OCLN	0.9947±0.06263	1.103±0.0271	1.043±0.08023
JAMA	0.9483±0.1035	1.04±0.02233	1.06±0.02849
TJP1	1.066±0.06749	0.8833±0.04955	1.039±0.06136
TJP2	1.035±0.04856	1.108±0.02307	1.108±0.04109
TJP3	1.063±0.02241	1.035±0.0735	0.9973±0.05277
CDH1	0.9108±0.06434	0.9594±0.02034	0.9582±0.06263
CTNNB1	0.9882±0.06742	1.157±0.01895	1.076±0.0542
NOX1	1.044±0.01637	1.014±0.008253	0.9137±0.04268
ALOX5	0.9145±0.1686	1.138±0.02137	1.169±0.01953
SOD1	1.586±0.3198	1.324±0.01466	1.254±0.008716
SOD2	1.191±0.2018	1.109±0.02795	1.008±0.06145
SOD3	0.878±0.002138	1.232±0.02815	1.024±0.09579

Table S4. Relative quantification (±SEM) of gene expressions in HT-29 after treatment of DINP fermenter extracts.

	Relative quantification		
**Gene**	DINP-D1	DINP-D2	DINP-D3
CLDN1	0.8531±0.04828	0.9455±0.01522	0.9019±0.09038
CLDN2	1.036±0.03587	0.6696±0.002891	0.7632±0.0156
CLDN4	1.056±0.01266	0.8953±0.08368	0.7127±0.03415
CLDN7	1.076±0.01897	0.9582±0.01674	1.007±0.02786
OCLN	0.9685±0.05839	0.8757±0.022	0.9031±0.00286
JAMA	1.044±0.02772	0.8751±0.02612	0.8529±0.05331
TJP1	0.857±0.065	0.8334±0.07576	0.5728±0.07238
TJP2	1.048±0.01015	0.9657±0.01815	1.009±0.006424
TJP3	1.06±0.008067	0.9495±0.03989	0.9312±0.02053
CDH1	1.039±0.04325	0.8784±0.02487	0.8043±0.04007
CTNNB1	1.08±0.01597	0.9883±0.03492	1.011±0.01365
NOX1	0.9194±0.04003	0.9548±0.07252	0.8301±0.0651
ALOX5	1.059±0.022	0.8772±0.03368	0.9102±0.05305
SOD1	1.157±0.014	1.03±0.02049	1.07±0.001907
SOD2	0.7844±0.06541	0.7032±0.03073	0.6117±0.05222
SOD3	1.37±0.05954	0.8727±0.1476	0.4493±0.08947

Table S5. Relative quantification (±SEM) of gene expressions in RAW264.7 after treatment of DINP fermenter extracts.

	**Relative quantification**		
**Gene**	DEHP-D1	DEHP-D2	DEHP-D3
TNFA	1.034±0.1169	0.9996±0.09255	1.057±0.03531
IL1B	0.9649±0.07171	0.8395±0.07499	0.9481±0.03815
IL6	1.119±0.1741	0.9839±0.07372	1.124±0.02223
KC	1.157±0.1467	0.9007±0.02132	1.055±0.03667
IL12p40	1.263±0.2761	0.8972±0.04101	0.8852±0.01306
IL23p19	1.167±0.2451	0.9479±0.006882	0.9298±0.019
IL10	1.172±0.2084	1.012±0.02511	0.9021±0.03905
IL10R1	1.269±0.2419	1.089±0.0261	1.007±0.05883
IL10R2	1.329±0.2299	1.084±0.01746	1.014±0.03005
IL1RA	1.085±0.07109	1.001±0.04746	1.045±0.02085
TGFB1	1.313±0.2503	0.9266±0.1387	0.9465±0.05713
IRF4	1.189±0.05187	1.039±0.03973	1.143±0.02448
SOCS3	0.7903±0.02948	0.7077±0.02544	0.7546±0.05669
MRC1	0.9654±0.04383	0.9644±0.02119	0.8408±0.1028
TGM2	1.161±0.06351	1.016±0.04839	1.033±0.05564

Table S6. Relative quantification (±SEM) of gene expressions in RAW264.7 after treatment of DINP fermenter extracts.

	Relative quantification		
**Gene**	DINP-D1	DINP-D2	DINP-D3
TNFA	0.9965±0.008059	1.042±0.0877	1.018±0.06159
IL1B	0.9067±0.0248	1.02±0.05013	1.034±0.04679
IL6	0.6468±0.04331	0.9738±0.07437	1.006±0.05169
KC	0.846±0.02742	0.9765±0.1305	0.9795±0.06823
IL12p40	1.157±0.01854	1.131±0.1089	1.19±0.001426
IL23p19	1.108±0.01215	1.061±0.02128	1.001±0.05446
IL10	0.7251±0.04831	0.8503±0.03862	0.7713±0.03022
IL10R1	1.018±0.02961	0.8884±0.1141	0.9173±0.09203
IL10R2	0.8498±0.02598	0.8825±0.04977	0.8244±0.01304
IL1RA	0.8116±0.01026	0.8726±0.0264	0.8281±0.01964
TGFB1	0.9961±0.0497	1.069±0.06971	0.989±0.07177
IRF4	0.755±0.01856	0.7306±0.07045	0.7606±0.04941
SOCS3	0.6854±0.05489	0.7603±0.1543	0.6685±0.06243
MRC1	0.9308±0.06237	0.9651±0.06632	0.8192±0.078
TGM2	0.8833±0.08747	0.6965±0.09232	0.6256±0.06459
